# Modular Relaxed Dependencies in Weak Memory Concurrency

**DOI:** 10.1007/978-3-030-44914-8_22

**Published:** 2020-04-18

**Authors:** Marco Paviotti, Simon Cooksey, Anouk Paradis, Daniel Wright, Scott Owens, Mark Batty

**Affiliations:** ETH Zurich, Zurich, Switzerland; 9 Imperial College London, London, UK; 10 University of Kent, Canterbury, UK; 11 ETH Zurich, Zürich, Switzerland

**Keywords:** Thin-air problem, Weak memory concurrency, Compiler Optimisations, Denotational Semantics, Compositionality

## Abstract

We present a denotational semantics for weak memory concurrency that avoids *thin-air reads*, provides data-race free programs with sequentially consistent semantics (DRF-SC), and supports a compositional refinement relation for validating optimisations. Our semantics identifies false program dependencies that might be removed by compiler optimisation, and leaves in place just the dependencies necessary to rule out thin-air reads. We show that our dependency calculation can be used to rule out thin-air reads in any axiomatic concurrency model, in particular C++. We present a tool that automatically evaluates litmus tests, show that we can augment C++ to fix the thin-air problem, and we prove that our augmentation is compatible with the previously used compilation mappings over key processor architectures. We argue that our dependency calculation offers a practical route to fixing the longstanding problem of thin-air reads in the C++ specification.

### 1 Introduction

It has been a longstanding problem to define the semantics of programming languages with shared memory concurrency in a way that does not allow unwanted behaviours – especially observing *thin-air* values [8,7] – and that does not forbid compiler optimisations that are important in practice, as is the case with Java and Hotspot [30,29]. Recent attempts [16,11,25,15] have abandoned the style of *axiomatic models*, which is the de facto paradigm of industrial specification [8,2,6]. Axiomatic models comprise rules that allow or forbid individual program executions. While it is impossible to solve all of the problems in an

* This work was funded by EPSRC Grants EP/M017176/1, EP/R020566/1 and EP/S028129/1, the Lloyds Register Foundation, and the Royal Academy of Engineering.

axiomatic setting [7], abandoning it completely casts aside mature tools for automatic evaluation [3], automatic test generation [32], and model checking [23], as well as the hard-won refinements embodied in existing specifications like C++, where problems have been discovered and fixed [8,7,18]. Furthermore, the industrial appetite for fundamental change is limited. In this paper we offer a solution to the thin-air problem that integrates with existing axiomatic models.

The thin-air problem in C++ stems from a failure to account for dependencies [22]: *false dependencies* are those that optimisation might remove, and *real dependencies* must be left in place to forbid unwanted behaviour [7]. A single execution is not sufficient to discern real and false dependencies. A key insight from previous work [14,15] is that event structures [33,34] give us a simultaneous overview of all traces at once, allowing us to check whether a write is sure to happen in every branch of execution. Unfortunately, previous work does not integrate well with axiomatic models, nor lend itself to automatic evaluation.

To address this, we construct a denotational semantics in which the meaning of an entire program is constructed by combining the meanings of its subcomponents via a compositional function over the program text. This approach can be particularly amenable to automatic evaluation, reasoning and compiler certification [19,24], and fits with the prevailing axiomatic approach.

This paper uses this denotational approach to capturing program dependencies to explore the thin-air problem, resulting in a concrete proposal for fixing the thin-air problem in the ISO standard for C++.

*Contributions.* There are two parts to the paper. In the first, we develop a denotational model called "Modular Relaxed Dependencies model" (MRD) and build metatheory around it. The model uses a relatively simple account of synchronisation, but it demonstrates separation between the calculation of dependency and the enforcement of synchronisation. In the second, we evaluate the dependency calculation by combining it with the fully-featured axiomatic models RC11 [18] and IMM [26].

The denotational semantics has the following advantages:

1. It is the first thin-air solution to support fork/join (§2.2).
2. It satisfies the DRF-SC property for a compositional model (§5): programs without data races behave according to sequential consistency.
3. It comes with a refinement relation that validates program transformations, including the optimisation that makes Hotspot unsound for Java [30,29], and a list of others from the Java Causality Tests [27] (§7).
4. It is shown to be equivalent to a global semantics that first performs a dependency calculation and then applies an axiomatic model.
5. An example in Section 10 illustrates a case in which thin-air values are observable in the current state-of-the-art models but forbidden in ours.

We adopt the dependency calculation from the global semantics of point 4 as the basis of our C++ model, which we call MRD-C11. We establish the C++ DRF-SC property described in the standard [13] (§9.1) and we provide several desirable properties for a solution to the thin-air problem in C++:

5. We show that our dependency calculation is the first that can be applied to any axiomatic model, and in particular the RC11 and IMM models that cover C++ concurrency (§8).
6. Our augmented IMM model, which we call MRD+IMM, is provably implementable over x86, Power, ARMv8, ARMv7 and RISC-V, with the compiler mappings provided by the IMM [26] (§8.1).
7. These augmented models of C++ are the first that solve the thin-air problem to have a tool that can automatically evaluate litmus tests (§11).

#### 1.1 Modular Relaxed Dependency by example

To simplify things for now, we will attach an `Init` program to the beginning of each example to initialise all global variables to zero. Doing this makes the semantics non-compositional, but it is a natural starting place and aligns well with previous work in the area. Later, after we have made all of our formal definitions, we will see why the `Init` program is not necessary.

For now, consider a simple programming language where all values are booleans, registers (ranging over `r`) are thread-local, and variables (ranging over `x`, `y`) are global. Informally, an event structure for a program consists of a directed graph of events. Events represent the global variable reads and writes that occur on all possible paths that the program can take. This can be built up over the program as follows: each write generates a single event, while each read generates two – one for each possible value that could be read. These read events are put in *conflict* with each other to indicate that they cannot both happen in a single execution, this is indicated with a zig-zag red arrow between the two events. Additionally, the event structure tracks true dependencies via an additional relation which we call *semantic dependencies* (DP). These are yellow arrows from read events to write events.

For example, consider the program

$$(\mathtt{r}_1 \mathtt{\ :=\ x;\ y\ :=\ } \mathtt{r}_1) \qquad (LB_1)$$

that reads from a variable `x` and then writes the result to `y`. The interpretation of this program is an event structure depicted as follows:

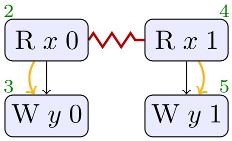

Each event has a unique identifier (the number attached to the box). The straight black arrows represent program order, the curved yellow arrows indicate a causal dependency between the reads and writes, and the red zigzag represents a conflict between two events. If two events are in conflict, then their respective continuations are in conflict too.

If we interpret the program `Init;` $LB_1$, as below, we get a program where the `Init` event sets the variables to zero.

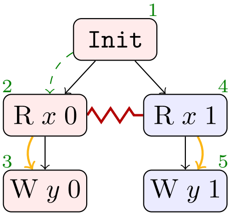

In the above event structure, we highlight events $\{1, 2, 3\}$ to identify an execution. The green dotted arrow indicates that event 2 reads its value from event 1, we call this relation *reads-from* (RF). This execution is *complete* as all of its reads read from a write and it is closed w.r.t conflict-free program order.

We interpret the following program similarly,

$$(\texttt{r}_2 \texttt{ := y; x := r}_2) \qquad (LB_2)$$

leading to a symmetrical event structure where the write to `x` is dependent on the read from `y`.

The interpretation of `Init;` $(LB_1 \parallel LB_2)$ gives the event structure where $(LB_1)$ and $(LB_2)$ are simply placed alongside one another.

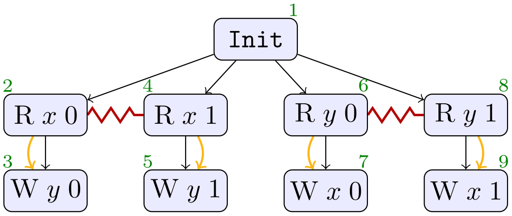

The interpretation of parallel composition is the union of the event structures from $LB_1$ and $LB_2$ without any additional conflict edges. When parallel composing the semantics of two programs, we add all RF-edges that satisfy a coherence axiom. Here we present an axiom that provides desirable behaviour in this example (Section 4 provides our model's complete axioms).

$$(\text{DP} \cup \text{RF}) \text{ is acyclic}$$

The program `Init;` $(LB_1 \parallel LB_2)$ allows executions of the following three shapes.

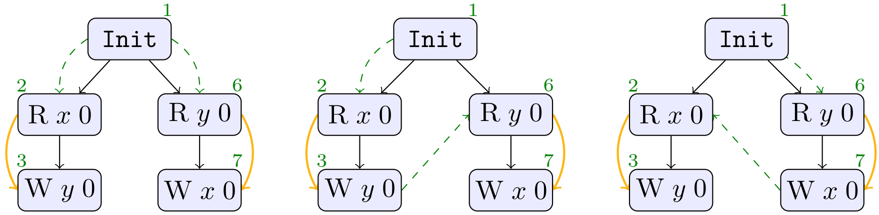

Note that in this example, we are not allowed to read the value 1 – reading a value that does not appear in the program is one sort of thin-air behaviour, as described by Batty et al. [7]. For example, the execution $\{1, 4, 5, 8, 9\}$ does not satisfy the coherence axiom as $4 \xrightarrow{\text{DP}} 5 \xrightarrow{\text{RF}} 8 \xrightarrow{\text{DP}} 9 \xrightarrow{\text{RF}} 4$ forms a cycle.

We now substitute $(LB_2)$ with the following code snippet

$$\texttt{r}_1 \texttt{ := y; x := } 1 \qquad (LB_3)$$

where the value written to the variable `x` is a constant. Its generated event structure is depicted as follows

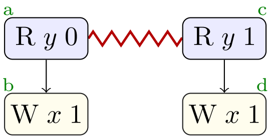

In this program, for each branch, we can reach a write of value 1 to location `x`. Hence, this will happen no matter which branch is chosen: we say $b$ and $d$ are *independent writes* and we draw no dependency edges from their preceding reads.

Consider now the program $(LB_3)$ in parallel with $LB_1$ introduced earlier in this section. As usual, we interpret the `Init` program in sequence with $(LB_1 \parallel LB_3)$ as follows:

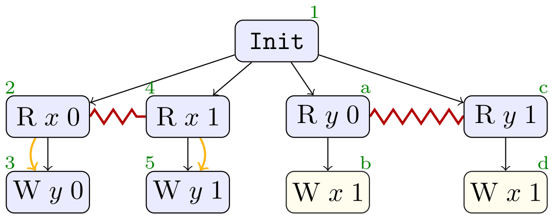

The resulting event structure is very similar to that of $(LB_1 \parallel LB_2)$, but the executions permitted in this event structure are different. The dependency edges calculated when adding the read are preserved, and now executions $\{1, 2, 3, a, b\}$ and $\{1, a, b, 4, 5\}$ are allowed. However, this event structure also contains the execution in which $d$ is independent.

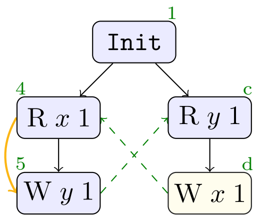

In the execution $\{d \xrightarrow{\text{RF}} 4 \xrightarrow{\text{DP}} 5 \xrightarrow{\text{RF}} c\}$ there is no RF or DP edge between $d$ and $c$ that can create a cycle, hence this is a valid complete execution in which we can observe $\texttt{x} = 1, \texttt{y} = 1$. Note that the `Init` is irrelevant in the consistency of this execution.

*Modularity.* It is worthwhile underlining the role that modularity plays here. In order to compute the behaviour of $(LB_1 \parallel LB_2)$ and $(LB_1 \parallel LB_3)$ we did not have to compute the behaviour of $LB_1$ again. In fact, we computed the semantics of $LB_1$, $LB_2$ and $LB_3$ in isolation and then we observed the behaviour in parallel composition.

*Thin-air values.* The program $(LB_1 \parallel LB_3)$ is a standard example in the weak memory literature called *load buffering*. In the program $(LB_1 \parallel LB_2)$, if event 5 or 9 were allowed in a complete execution, that would be an undesirable thin-air behaviour: there is no value 1 in the program text, nor does any operation in the program compute the value 1. The program $(LB_1 \parallel LB_3)$ is similar, but now contains a write of value 1 in the program text, so this is no longer a thin-air value. Note that the execution given for it is not sequentially consistent, but nonetheless a weak memory model needs to allow it so that a compiler can, for example, swap the order of the two commands in $LB_3$, which are completely independent of each other from its perspective.

### 2 Event Structures

Event structures will form the semantic domain of our denotational semantics in Section 5. Our presentation follows the essential ideas of Winskel [33] and is further influenced by the treatment of shared memory by Jeffrey and Riely [15].

#### 2.1 Background

A partial order $(E, \sqsubseteq)$ is a set $E$ equipped with a reflexive, transitive and antisymmetric relation $\sqsubseteq$. A well-founded partial order is a partial order that has no infinite decreasing chains of the form $\cdots \sqsubseteq e_{i-1} \sqsubseteq e_i \sqsubseteq e_{i+1} \cdots$.

A *prime event structure* is a triple $(E, \sqsubseteq, \#)$. $E$ is a set of events, $\sqsubseteq$ is a well-founded partial order on $E$ and $\#$ is a conflict relation on $E$. $\#$ is binary, symmetric and irreflexive such that, for all $c, d, e \in E$, if $c \# d \sqsubseteq e$ then $c \# e$. We write $\mathsf{Con}(E)$ for the set of *conflict-free* subsets of $E$, *i.e.* those subsets $C \subseteq E$ for which there is no $c, d \in C$ such that $c \# d$.

*Notation.* We use $E$ to range over (prime/labelled/memory) event structures, and also the event set contained within, when there is no ambiguity. We also use $\mathcal{E}$ for event structures.

A *labelled event structure* $(E, \sqsubseteq, \#, \lambda)$, over a set of labels $\Sigma$, is a prime event structure together with a function $\lambda : E \to \Sigma$ which assigns a label to an event. We make events explicit using the notation $\{e : \sigma\}$ for $\lambda(e) = \sigma$. We sometimes avoid using names and just write the label $\sigma$ when there is no risk of confusion.

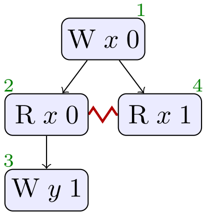

Consider the labelled event structure formed by the set $\{1, 2, 3, 4\}$, where the order relation is defined such that $1 \sqsubseteq 2 \sqsubseteq 3$ and $1 \sqsubseteq 4$, the conflict relation is defined such that $2\#4$ and $3\#4$, and the labelling function is defined such that $\lambda(1) = (\mathrm{W}\ x\ 0)$, $\lambda(2) = (\mathrm{R}\ x\ 0)$, $\lambda(3) = (\mathrm{W}\ y\ 1)$ and $\lambda(4) = (\mathrm{R}\ x\ 1)$. The event structure is visualised on the left (we elide conflict edges that can be inferred from order).

Given labelled event structures $\mathcal{E}_1$ and $\mathcal{E}_2$ define the *product* labelled event structure $\mathcal{E}_1 \times \mathcal{E}_2 \triangleq (E, \sqsubseteq, \#, \lambda)$. $E$ is $E_1 \cup E_2$, assuming $E_1$ and $E_2$ to be disjoint, $\sqsubseteq$ is $\sqsubseteq_1 \cup \sqsubseteq_2$, $\#$ is $\#_1 \cup \#_2$ and $\lambda$ is $\lambda_1 \cup \lambda_2$.

The *coproduct* labelled event structure $\mathcal{E}_1 + \mathcal{E}_2$ is the same as the product, except that the conflict relation $\#$ is $\#_1 \cup \#_2 \cup \{E_1 \times E_2\} \cup \{E_2 \times E_1\}$. We can use a similar construction for the co-product of an infinite set of pairwise-disjoint labelled event structures, indexed by $I$: we take infinite unions on the underlying sets and relations, along with extra conflicts for every pair of indices. Where the $\mathcal{E}_i$ are not disjoint, we can make them so by renaming with fresh event identifiers. In particular, we will need the infinite coproduct $\sum_{i \in I} \mathcal{E}$ with as many copies of $\mathcal{E}$ as the cardinality of the set $I$, and all the events between each copy in conflict. Each of these copies will by referred to as $\mathcal{E}^i$.

For a labelled event structure $\mathcal{E}_0$ and an event $e$, where $e \notin E_0$, define the *prefix* labelled event structure, $e \bullet \mathcal{E}_0$, as a labelled event structure $(E, \sqsubseteq, \#, \lambda)$ where $E$ equals $E_0 \cup \{e\}$, $\sqsubseteq$ equals $\sqsubseteq_0 \cup (\{e\} \times E)$, and $\#$ equals $\#_0$.

#### 2.2 The fork-join event structure

Our language supports parallel composition nested under sequential composition, so we will need to model spawning threads and a subsequent wait for their termination. To support this, we define the *fork-join* composition of two labelled event structures, $\mathcal{E}_1 \star \mathcal{E}_2$. First we define the leaves, $\downarrow(\mathcal{E})$, as the $\sqsubseteq$-maximal elements of $\mathcal{E}$. Let $I$ be the set of maximal conflict-free subsets of $\downarrow(\mathcal{E}_1)$. Intuitively, each event set in $I$ corresponds to the last events[4] of one way of executing the concurrent threads in $\mathcal{E}_1$. We then generate a fresh copy of $\mathcal{E}_2$ for each of the executions: $\mathcal{E}_3 = \sum_{i \in I} \mathcal{E}_2$.

Now $\mathcal{E}_1 \star \mathcal{E}_2 \triangleq (E, \sqsubseteq, \#, \lambda)$ such that $E$ is $E_1 \cup E_3$, $\#$ is $\#_1 \cup \#_3$, $\lambda$ is $\lambda_1 \cup \lambda_3$, $\sqsubseteq$ is the transitive closure of

$$\sqsubseteq_1 \cup \sqsubseteq_3 \cup \bigcup_{i \in I} \{(e, e') \mid e \in i \wedge e' \in \mathcal{E}_2^E\}$$

The set of events, $E$, is the set $E_1$ plus all the elements from the copies of $E_3$. The order, $\sqsubseteq$, is constructed by linking every event in the copy $\mathcal{E}_2^i$, with all the events in the set $i$, plus the obvious order from $E_1$ and the order in the local copy $\mathcal{E}_2^i$. Finally, the conflict relation is the union of the conflict in $\mathcal{E}_1$ and $\mathcal{E}_3$.

### 3 Coherent event structure

The signature of labels, $\Sigma$, is defined as follows:

$$\Sigma = (\{\mathrm{R}, \mathrm{W}\} \times \mathcal{X} \times \mathcal{V}) + \{\mathrm{L}\} + \{\mathrm{U}\}$$

where $(\mathrm{W}\ x\ v) \in \Sigma$ and $(\mathrm{R}\ x\ v) \in \Sigma$ are the usual write and read operations and L, U are the lock and unlock operations respectively.

A *coherent event structure* is a tuple $(E, S, \vdash, \leq)$ where $E$ is a labeled event structure. $S$ is a set of *partial executions*, where each execution is a tuple comprising a maximal conflict-free set of events, together with an intra-thread reads-from

[4] We assume that there are no infinite increasing $\sqsubseteq$-chains in $\mathcal{E}_1$.

relation $\text{RF}_i$, an extra-thread reads-from $\text{RF}_e$, a dependency relation $\text{DP}$, and a *partial order* on lock/unlock events $\text{LK}$. The justification relation, $\vdash$, is a relation between conflict-free sets and events. Finally, the *preserved program order*, $\leq^{\mathcal{X}}$, is a restriction of the program order, $\sqsubseteq$, for events on the same variable. $\leq^{\text{L}}$ is the restriction of program order on events related in program order with locks or unlocks. Finally, we define $\text{RF}$ to be $\text{RF}_e \cup \text{RF}_i$ and $\leq$ to be $\leq^{\mathcal{X}} \cup \leq^{\text{L}}$. For a partial execution, $X \in S$, we denote its components as $\text{LK}_X$, $\text{RF}_X$ and $\text{DP}_X$.

Justification, $\vdash$, collects dependency information in the program and is used to calculate $\text{DP}_X$. For a conflict-free set $C$ and an event $e$, we say $C$ *justifies* $e$ or $e$ *depends* on $C$ whenever $C \vdash e$. We collect dependencies between events modularly in order to identify the so-called independent writes which will be introduced shortly.

For a given partial execution, $X$, we define the order $\text{HB}_X$ as the reflexive transitive closure of $(\sqsubseteq \cup \text{LK}_X)$. A coherent event structure contains a *data race* if there exists an execution $X$, with two events on the same variable $x$, at least one of which is a write, that are not ordered by $\text{HB}_X$. A coherent event structure is *data-race-free* if it does not contain any data race. A *racy* $\text{RF}_X$*-edge* is when two events $w$ and $r$ are racy and $w \xrightarrow{\text{RF}_e}_X r$. Note that $\text{RF}_i$ edges cannot ever be racy. We now define a coherent partial execution.

**Definition 1 (Coherent Partial Execution).** *A partial execution $X$ is* coherent *if and only if:*

1. $(\leq^{\text{L}} \cup \text{LK}_X \cup \text{DP}_X \cup \text{RF}_{eX})$ *is acyclic, and*
2. *if* $(w : \text{W}\ x\ v) \xrightarrow{\text{RF}}_X (r : \text{R}\ x\ v)$ *there are no* $(e : \text{R}\ x\ v')$ *or* $(e : \text{W}\ x\ \_)$ *such that* $w \xrightarrow{\text{HB}_X} e \xrightarrow{\text{HB}_X} r$ *with* $v \neq v'$.

A *complete execution* $X$ is an execution where all read events $r$ have a write $w$ that they read from, i.e. $w \xrightarrow{\text{RF}}_X r$.

### 4 Weak memory model

Central to the model is the way it records program dependencies in $\vdash$ and $\text{DP}$. Justification, $\vdash$, records the structure of those dependencies in the program that may be influenced by further composition. As we shall see, composing programs may add or remove dependencies from justification: for example, composing a read may make later writes dependent, or the coproduct mechanism, introduced shortly, may remove them. In some parts of the program, e.g. inside locked regions, dependencies do not interact with the context. In this case, we *freeze* the justifications, using them to calculate $\text{DP}$. Following a freeze, the justification relation is redundant and can be forgotten – $\text{DP}$ can be used to judge which executions are coherent.

*Freezing.* Here we define a function *freeze* which takes a justification $C \vdash (w : \text{W}\ x\ v)$ and gives the corresponding dependency relation $(r : \text{R}\ x\ v) \xrightarrow{\text{DP}} (w : \text{W}\ x\ v)$ iff $r \in C$. We lift *freeze* to a function on an event structure as follows:

$$freeze(E_1, S_1, \vdash_1, \leq_1) \triangleq (E_1, S, \emptyset, \leq_1) \quad (1)$$

where $S$ contains all the executions

$$(X_1, \text{LK}_{X_1}, (\text{DP}_{X_1} \cup \text{DP}), \text{RF}_{X_1})$$

where for each write, $w_i \in X_1$, we choose a justification so that $C_1 \vdash_1 w_1, ..., C_n \vdash_1 w_n$ covers all writes in $X_1$. Furthermore, with DP defined as follows:

$$\text{DP} = (\bigcup_{i \in \{1, \cdots n\}} freeze(C_i \vdash w_i))$$

$X_1$ must be a *coherent execution*. We prove that for a coherent execution there always exists a choice of write justifications that freeze into dependencies to form a coherent execution.

We will illustrate freezing of the program,

$$\mathtt{r}_1 \mathtt{:=x;\ r}_2 \mathtt{:=t;}\ \textbf{if}\mathtt{(r}_1 == 1 \vee \mathtt{r}_2 == 1\mathtt{)\{y := }1\}$$

whose event structure is as follows:

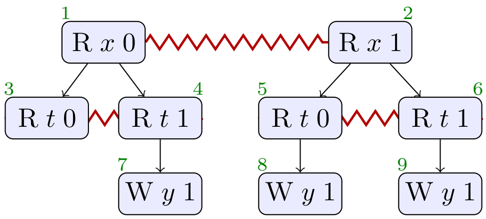

The rules later on in this section will provide us with justifications $\{(6 : \text{R}\ t\ 1)\} \vdash (9 : \text{W}\ y\ 1)$ and $\{(2 : \text{R}\ x\ 1)\} \vdash (9 : \text{W}\ y\ 1)$ (but not the *independent justification* $\vdash (9 : \text{W}\ y\ 1)$). So in this program there are two *minimal* justifications of $(9 : \text{W}\ y\ 1)$. The result of freezing is to duplicate all partial executions for each choice of write justifications. In this case, we get an execution containing $2 \xrightarrow{\text{DP}} 9$ and another one containing $6 \xrightarrow{\text{DP}} 9$.

### 4.1 Prepending single events

When prepending loads and stores, we model forwarding optimisations by updating the justification relation: e.g. when prepending a write, (w : W x 0), to an event structure where $\{(r : \text{R}\ x\ 0)\} \vdash w'$, write forwarding satisfies the read of the justification, leaving an independently justified write, $\vdash w'$.

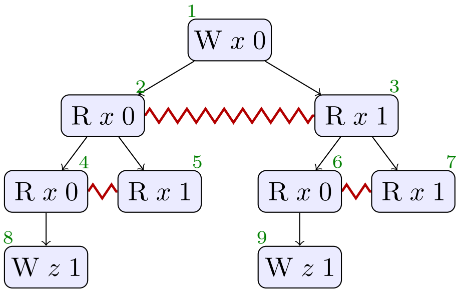

Forwarding is forbidden if there exists $e$ in $E$ such that $w \leq e \leq r$, as in the example on the left. In this example we do not forward 1 to 6. The rules of this section give us that $\{1, 3, 6\} \vdash 9$: we have preserved program order over the accesses of $x$, $1 \leq 3 \leq 6$, and we do not forward across the intervening read 3.

**Read Semantics** We now define the semantics of read prepending as follows:

$$(r : \mathrm{R}\ x\ v) \bullet (E_1, S_1, \vdash_1, \leq_1) \triangleq ((r : \mathrm{R}\ x\ v) \bullet E_1, S, \vdash, \leq) \tag{2}$$

where preserved program order $\leq$ is built straightforwardly out of $\leq_1$, ordering locks, unlocks and same-location accesses, and $S$ is defined as the set of all $(X \cup \{r\}, \mathrm{LK}_X, \mathrm{RF}_X, \mathrm{DP}_X)$, where $X$ is a partial execution of $S_1$ and $\vdash$ is the smallest relation such that for all $C \vdash_1 e$ we have

$$C_1 \cup \{r\} \setminus \mathrm{LF} \vdash e$$

with LF being the "*Load Forwarded*" set of reads, *i.e.*the set of reads consecutively following the matching prepended one:

$$\mathrm{LF} = \{(r' : \mathrm{R}\ x\ v) \in C_1 \mid \nexists e', r \leq^{\mathcal{X}} e' \leq^{\mathcal{X}} r'\}$$

This allows for load forwarding optimisations and coherence is satisfied by construction.

**Write Semantics** The write semantics are then defined as follows:

$$(w : \mathrm{W}\ x\ v) \bullet (E_1, S_1, \vdash_1, \leq_1) \triangleq ((w : \mathrm{W}\ x\ v) \bullet E_1, S, \vdash, \leq) \tag{3}$$

where $\leq$ is built as in the read rule and $S$ contains all *coherent* executions of the form,

$$(X \cup \{w\}, \mathrm{LK}_X, (\mathrm{RF}_X \cup \mathrm{RF}_i), \mathrm{DP}_X)$$

where $X \in S_1$, and $w \xrightarrow{\mathrm{RF}_i} r$ for any set of matching reads $r$ in $E_1$ such that condition (1.2) of coherence is satisfied. Adding $\mathrm{RF}_i$ edges leaves condition (1.1) satisfied.

The justification relation $\vdash$ is the smallest upward-closed relation such that for all $C \vdash_1 e$:

1. $\vdash w$
2. $C \setminus \mathrm{SF} \cup \{w\} \vdash e$ if there exists $e' \in C$ s.t. $w \leq^{\mathcal{X}} e'$
3. $C \setminus \mathrm{SF} \vdash e$ otherwise with SF being the *Store Forwarding* set of reads, *i.e.*the set of reads that we are going to remove from the justification set for later events that are matching the write we are prepending. This is defined as follows:

$$\mathrm{SF} = \{(r' : \mathrm{R}\ x\ v) \mid\ \nexists e, w \leq^{\mathcal{X}} e \leq^{\mathcal{X}} r'\}$$

When prepending a write to an event structure, we add it to justifications that contain a read to the same variable. Failing to do so would invalidate the DRF-SC property. We provide an example in Section 6.3, but we need to complete the definition of the semantics first, in particular, we need to explain first how the writes are lifted. This is coming in the next section (Section 4.2).

### 4.2 Coproduct semantics

The coproduct mechanism is responsible for making writes independent of prior reads if they are sure to happen, regardless of the value read. It produces the independent writes that enabled relaxed behaviour in the example in Section 1.

In the definition of coproduct we use an upward-closure of justification to enable the lifting of more dependencies. Whenever $C \vdash e$ we define $\uparrow(C)$ as the upward-closed justification set, i.e. $D \vdash e$ if $C \vdash e$, $D$ is a conflict-free lock-free set with $C \subseteq D$, such that for all $e' \in D$ if $e''$ is an event such that $e'' \leq e'$ then $e'' \in D$.

Now we define the coproduct operation. If $E_1$ is a labelled event structure of the form $(r_1 : \mathrm{R}\ x\ v_1) \bullet E_1'$ and, similarly, $E_2$ is of the form $(r_2 : \mathrm{R}\ x\ v_2) \bullet E_2'$, the coproduct of event structures is defined as,

$$(E_1, S_1, \vdash_1, \leq_1) + (E_2, S_2, \vdash_2, \leq_2) \triangleq (E_1 + E_2, S_1 \cup S_2, (\vdash_1 \cup \vdash_2 \cup \vdash), \leq)$$

where whenever $\{r_1\} \cup C_1 \vdash_1 (w : \mathrm{W}\ y\ v)$ and $\{r_2\} \cup C_2 \vdash_2 (w' : \mathrm{W}\ y\ v)$ then if the following conditions hold, we have $D' \vdash w$ and $D'' \vdash w'$:

1. there exists a $D' \in \uparrow(C_1)$ that is isomorphic to a $D'' \in \uparrow(C_2)$, that is, there exists $f : D' \to D''$ that is a $\lambda$-preserving and $\leq^{\mathcal{X}}$-preserving bijection,
2. there is no event $e$ in $D'$ such that $r_1 \leq^{\mathcal{X}} e$

The example of Section 1 illustrates the application of condition (1) of coproduct. Recall the event structures of $(LB_1)$ and $(LB_3)$ respectively.

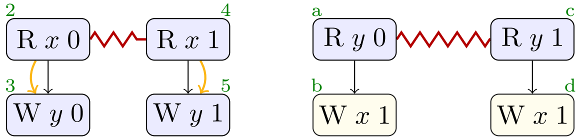

In each case, the event structure is built as the coproduct of the conflicting events. In $(LB_3)$, prior to applying coproduct we have $\{a\} \vdash b$ and $\{c\} \vdash d$. The writes have the same label for both read values so, taking $C_1$ and $C_2$ to be empty, coproduct makes them independent, adding the independent writes $\vdash b$ and $\vdash d$.

In contrast, the values of writes 3 and 5 differ in $(LB_1)$, so the coproduct has $\{2\} \vdash 3$ and $\{4\} \vdash 5$. When ultimately frozen, the justifications of $(LB_1)$ will produce the dependency edges $(2,3)$ and $(4,5)$ as described in Section 1.

As for condition (2), if there is an event in the justification set that is ordered in $\leq^{\mathcal{X}}$ with the respective top read, then the top read cannot be erased from the justification. Doing so would break the $\leq^{\mathcal{X}}$ link.

When having value sets that contain more than two values, we use $\sum_{v\in\mathcal{V}}$ to denote a *simultaneous coproduct* (rather than the infinite sum). More precisely, if we coproduct the event structures $E_0, E_1, \cdots, E_n$ in a pairwise fashion as follows,

$$(\cdots(E_0 + E_1) + \cdots) + E_v$$

we would get liftings that are undesirable. To see this, it suffices to consider the program,

$$\textbf{if}\,(\texttt{r==3})\{\texttt{x := 2}\}\{\texttt{x := 1}\}$$

where the write to x of 1 is independent for a coproduct over values 1 and 2, but not when considering the event structure following (R $x$ 3).

#### 4.3 Lock semantics

When prepending a lock, we order the lock before following events in $\leq$ and we freeze the justifications into dependencies. By freezing, we prevent justifications from events after the lock from interacting with newly appended events. This disables optimisations across the lock, e.g. store and load forwarding.

We define the semantics of locks as follows,

$$(l : \mathrm{L}) \bullet (E_1, \vdash_1, S_1, \leq_1) \triangleq ((l : \mathrm{L}) \bullet E_1, \emptyset, S, \leq) \tag{4}$$

where $\leq^{\mathcal{X}}$ remains unchanged and $(E_1', \emptyset, S_1', \leq_1') = \mathit{freeze}(E_1, \vdash_1, S_1, \leq_1)$, where $S$ contains all partial executions of the form,

$$(X \cup \{l\}, (\mathrm{LK}_X \cup \mathrm{LK}), \mathrm{DP}_X, \mathrm{RF}_X)$$

where $X \in S_1'$ and the lock order LK is such that for all lock or unlock event $l' \in X$, $l \xrightarrow{\mathrm{LK}} l'$. Finally, $\leq^{\mathrm{L}}$ is $\leq^{\mathrm{L}}\,{}'_1$ extended with the lock ordered before all events in $E_1'$.

The semantics for the unlock is similar.

#### 4.4 Parallel composition

We define the parallel semantics as follows. Note that this operation freezes the constituent denotations before combining them, erasing their respective justification relations. This choice prevents the optimisation of dependencies across forks and it makes thread inlining optimisations unsound, as they are in the Promising Semantics [16] and the Java memory model [21].

$$(E_1, S_1, \vdash_1, \leq_1) \times (E_2, S_2, \vdash_2, \leq_2) \triangleq (E_1 \times E_2, S, \emptyset, \leq_1 \cup \leq_2)$$

where, $S$ are all *coherent* partial executions of the form,

$$(X_1 \cup X_2, (\text{LK}_{X_1} \cup \text{LK}_{X_2} \cup \text{LK}), (\text{DP}_{X_1} \cup \text{DP}_{X_2}), (\text{RF}_{X_1} \cup \text{RF}_{X_2} \cup \text{RF}_e))$$

where $X_1 \in S_1^F$, $X_2 \in S_2^F$ and

- $\mathit{freeze}(E_1, S_1, \vdash_1, \leq_1) = (E_1, S_1^F, \emptyset, \leq_1)$
- $\mathit{freeze}(E_2, S_2, \vdash_2, \leq_2) = (E_2, S_2^F, \emptyset, \leq_2)$

Furthermore, LK is constrained so that $(\text{LK}_{X_1} \cup \text{LK}_{X_2} \cup \text{LK})$ is a *total* order over the lock/unlock operations such that no lock/unlock operation is introduced between a lock and the next unlock on the same thread. Finally, we add all $(w : \text{W } x\ v) \xrightarrow{\text{RF}_e} (r : \text{R } x\ v)$ edges such that the execution satisfies condition (1.1) of coherence[1] and such that $w$ belongs to $S_1^F$ and $r$ belongs to $S_2^F$ or vice versa.

#### 4.5 Join Semantics

We define the join composition as follows:

$$(E_1, S_1, \vdash_1, \leq_1) \star (E_2, S_2, \vdash_2, \leq_2) \triangleq (E_1 \star E_2, S, \vdash_1, \leq) \quad (5)$$

where $\leq$ is built as in the read rule and $S$ are all executions of the form

$$(X_1 \cup X_2, (\text{LK}_{X_1} \cup \text{LK}_{X_2} \cup \text{LK}), (\text{DP}_{X_1} \cup \text{DP}_{X_2}), (\text{RF}_{X_1} \cup \text{RF}_{X_2} \cup \text{RF}_i))$$

where $X_1 \in S_1$ and $X_2 \in S_2$ with $X_1$ and $X_2$ conflict-free. Lock order LK orders all lock/unlock of $X_1$ before all lock/unlock of $X_2$ and $w \xrightarrow{\text{RF}_i} r$ whenever $w \in X_1$ and $r \in X_2$ such that the execution is still coherent.

### 5 Language and Semantics

We consider an imperative language that has sequential and parallel composition, and mutable shared memory.

**Definition 2 (Language).**

$$B := M = M \mid B \wedge B \mid B \vee B \mid \neg B \qquad M := n \mid \texttt{r}$$
$$P ::= \texttt{skip} \mid \texttt{r := x} \mid \texttt{x :=}\ M \mid P_1\texttt{;}\ P_2 \mid P_1 \parallel P_2 \mid \textbf{if}\texttt{(}B\texttt{)}\{P_1\}\{P_2\}$$
$$\mid \textbf{while}\texttt{(}B\texttt{)}\{P\} \mid \text{L} \mid \text{U}$$

We have standard boolean expressions, $B$, and expressions, $M$, represented by natural numbers, $n$, or registers, `r`. Finally we have the set of command statements, $P$, where `skip` is the command that performs no action, `r := x` reads from a global variable and stores the value in `r`, `x :=` $M$ computes the expression $M$ and stores its value to the global variable $x$, $P_1$`;` $P_2$ is sequential composition,

[1] Note that condition (1.2) does not need to be checked.

and $P_1 \parallel P_2$ is parallel composition. We have standard conditional statements, while loops, locks and unlocks. Moreover, a program $P$ is *lock-well-formed*[5] if on every thread, every lock is paired with a following unlock instruction and vice versa, and there is no lock or unlock operation between pairs.

A *register environment*, $\mathcal{R} \to \mathcal{V}$, is a function from the set of local registers, $\mathcal{R}$, to the set of values, $\mathcal{V}$. A *continuation* is a function taking a register environment, $\mathcal{R} \to \mathcal{V}$, to an event structure, $\mathcal{E}$. We write $\underline{\emptyset}$ as a short-hand for $\lambda\rho.\emptyset$, the continuation returning the empty event structure.

We interpret the syntax defined above into the semantic domain defined in Section 4. In Figure 1, we define $[\![\cdot]\!]$ as a function which takes a *step-index* $n$, a register environment $\rho$, and a continuation $\kappa$, and returns a coherent event structure.

The interpretation function $[\![\cdot]\!]$ is defined first by induction on the step-index and then by induction on the syntax of the program. When $n = 1$ the interpretation gives the empty event structure (undefined). Otherwise we proceed by induction on the structure of the program. `skip` is just the continuation applied to the environment. A read is interpreted as a set of conflicting read events for each value $v$ attached with a continuation applied to the environment where the register is updated with $v$.

A write is interpreted as a write with a following continuation. We interpret sequencing by interpreting the second program and passing it on to the interpretation of the first as a continuation. Parallel composition is the interpretation of the two programs with empty continuations passed to the $\times$ operator. The conditional statement is interpreted as usual. For interpreting the while-loops we use the induction hypothesis on the step-index [9].

When parallel composing two threads, we want to forbid any reordering with events sequenced before or after the composition (as thread inlining would do). To forbid this local reordering we surround this composition with two lock-unlock pairs.

#### 5.1 Compositionality

We define the language of contexts inductively in the standard way.

**Definition 3 (Context).**

$$\begin{aligned} \mathcal{C} ::= &\ [-] \mid P;\, \mathcal{C} \mid \mathcal{C};\ P \mid\ (\mathcal{C} \parallel P)\ \mid\ (P \parallel \mathcal{C}) \\ &\mid \textbf{if}\,(B)\{\mathcal{C}\}\{P\} \mid \textbf{if}\,(B)\{P\}\{\mathcal{C}\} \mid \textbf{while}\,(B)\{\mathcal{C}\} \end{aligned}$$

In the base case, the context is a hole, denoted by $[-]$. The inductive cases follow the structure of the program syntax. In particular, a context can be a program $P$ in sequence with a context, a context in sequence with a program $P$ and so on. For a context $\mathcal{C}$ we denote $\mathcal{C}[P]$ by the inductively defined function on the context $\mathcal{C}$ that substitutes the program $P$ in every hole.

[5] Jeffrey and Riely [15] adopt the same restriction. We conjecture that modelling blocking locks [4] would not affect the DRF-SC property.

$$\llbracket P \rrbracket_{1\ \rho\ \kappa} = \underline{\emptyset}$$
$$\llbracket \texttt{skip} \rrbracket_{n\ \rho\ \kappa} = \kappa(\rho)$$
$$\llbracket \texttt{r := x} \rrbracket_{n\ \rho\ \kappa} = \Sigma_{v \in V}(\mathrm{R}\ x\ v \bullet \kappa(\rho[r \mapsto v]))$$
$$\llbracket \texttt{x :=}\ M \rrbracket_{n\ \rho\ \kappa} = (\mathrm{W}\ x\ \llbracket M \rrbracket_\rho) \bullet \kappa(\rho)$$
$$\llbracket P_1\,;\ P_2 \rrbracket_{n\ \rho\ \kappa} = \llbracket P_1 \rrbracket_{n\ \rho\ (\lambda\rho. \llbracket P_2 \rrbracket_{n\ \rho\ \kappa})}$$

$$\llbracket \mathrm{L} \rrbracket_{n\ \rho\ \kappa} = (\mathrm{L} \bullet E_1, \vdash_1) \quad \text{where } (E_1, \vdash_1) = \kappa(\rho)$$
$$\llbracket \mathrm{U} \rrbracket_{n\ \rho\ \kappa} = (\mathrm{U} \bullet E_1, \vdash_1) \quad \text{where } (E_1, \vdash_1) = \kappa(\rho)$$

$$\llbracket P_1 \parallel P_2 \rrbracket_{n\ \rho\ \kappa} = \llbracket \mathrm{L}; \mathrm{U} \rrbracket_{n\ \rho\ \kappa'}$$
$$\text{where } \kappa' = (\lambda\rho.(\llbracket P_1 \rrbracket_{n\ \rho\ \underline{\emptyset}}) \times (\llbracket P_2 \rrbracket_{n\ \rho\ \underline{\emptyset}}) \star (\llbracket \mathrm{L};\ \mathrm{U} \rrbracket_{n\ \rho\ \kappa}))$$

$$\llbracket \textbf{if}\,(B)\,\{P_1\}\{P_2\} \rrbracket_{n\ \rho\ \kappa} = \begin{cases} \llbracket P_1 \rrbracket_{n\ \rho\ \kappa} & \llbracket B \rrbracket_\rho = \mathrm{T} \\ \llbracket P_2 \rrbracket_{n\ \rho\ \kappa} & \llbracket B \rrbracket_\rho = \mathrm{F} \end{cases}$$

$$\llbracket \textbf{while}\,(B)\,\{P\} \rrbracket_{n\ \rho\ \kappa} = \begin{cases} \llbracket P\,;\textbf{while}\,(B)\,\{P\} \rrbracket_{(n-1)\ \rho\ \kappa} & \llbracket B \rrbracket_\rho = \mathrm{T} \\ \llbracket \texttt{skip} \rrbracket_{n\ \rho\ \kappa} & \llbracket B \rrbracket_\rho = \mathrm{F} \end{cases}$$

Fig. 1: Semantic interpretation

The following lemma shows that the semantics preserve context application. This falls out from the fact that the semantic interpretation is compositional, that is, we define every constructor in terms of its subcomponents.

**Lemma 1 (Compositionality).** *For all programs $P_1$, $P_2$, if $\llbracket P_1 \rrbracket = \llbracket P_2 \rrbracket$ then for all contexts $\mathcal{C}$, $\llbracket \mathcal{C}[P_1] \rrbracket = \llbracket \mathcal{C}[P_2] \rrbracket$.*

The proof is a straightforward induction on the context $\mathcal{C}$ and it follows from the fact that semantics is inductively defined on the program syntax. The attentive reader may note that to prove $\llbracket P_1 \rrbracket = \llbracket P_2 \rrbracket$ in the first place we have to assume $n$, $\rho$ and $\kappa$ and prove $\llbracket P_1 \rrbracket_{n\ \rho\ \kappa} = \llbracket P_2 \rrbracket_{n\ \rho\ \kappa}$. It is customary however in denotational semantics to have programs denoted by functions that are equal if they are equal at all inputs [31].

#### 5.2 Data Race Freedom

Data race freedom ensures that we forbid optimisations which could lead to unexpected behaviour even in the absence of data races. We first define the *closed semantics* for a program $P$. For all $n$, the semantics of $P$, namely $\llbracket P \rrbracket$ is $\llbracket \texttt{Init}(P) \rrbracket_{n\ \lambda x.0\ \underline{\emptyset}}$, where $\texttt{Init}(P)$ is the program that takes the global variables in $P$ and initialises them to 0. We now establish that race-free programs interpreted in the closed semantics have sequentially consistent behaviour.

*DRF semantics.* Rather than proving DRF-SC directly, we prove that race-free programs behave according to an intermediate semantics $(\!|\cdot|\!)$. This semantics differs from $\llbracket \cdot \rrbracket$ in only two ways: program order is used in the calculation of coherence instead of preserved program order, and no dependency edges are recorded (as these are subsumed by program order). More precisely, the semantics is calculated as in Figure 1 but we check that $(\mathrm{RF}_e \cup \mathrm{LK} \cup \sqsubseteq)$ is acyclic.

Note that race-free executions of the intermediate semantics $(\!|\cdot|\!)$ satisfy the constraints of the model of Boehm and Adve [10], and the definition of race is the same between the two models. Boehm and Adve prove that in the absence of races, their model provides sequential consistency.

The DRF-SC theorem is stated as follows.

**Theorem 1.** *For any program $P$, if $(\!|P|\!)$ is data race free then every execution $D$ in $[\![P]\!]$ is a sequentially consistent execution,* i.e. *$D$ is in $(\!|P|\!)$.*

## 6 Tests and Examples

In this section, four examples demonstrate aspects of the semantics: the first recognises a false dependency, the second forbids unintended behaviour allowed by Jeffrey and Riely [15], the third motivates the choice to add forwarded writes to justification, and the last shows how we support an optimisation forbidden by Java but performed by the Hotspot compiler.

### 6.1 LB+ctrl-double

In the first example, from Batty et al. [7], the compiler collapses conditionals to transform $P_1$ to $P_2$.

| $P_1$ | | $P_2$ |
|---|---|---|
| $\mathtt{r_1}$ := x;<br>**if**($\mathtt{r_1}$==1){<br>  y := 1<br>} **else** {<br>  y := 1<br>} | $\longrightarrow$ | $\mathtt{r_1}$ := x;<br>y := 1 |

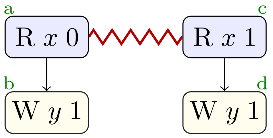

Coproduct ensures that the denotations of $P_1$ and $P_2$ are identical, with the event structure above, together with justification $\vdash b$ and $\vdash d$. From compositionality (Lemma 1) and equality of the denotations, we have equal behaviour of $P_1$ and $P_2$ in any context, and the optimisation is allowed.

### 6.2 Jeffrey and Riely's TC7

The next test is Java TC7. The outcome where $\mathtt{r_1}$, $\mathtt{r_2}$ and $\mathtt{r_3}$ all have value 1 is forbidden by Jeffrey and Riely [15, Section 7], but allowed in the Java Causality Test Cases [27].

| $T_1$ | | $T_2$ |
|---|---|---|
| $\mathtt{r_1}$ := z;<br>$\mathtt{r_2}$ := x;<br>y := $\mathtt{r_2}$ | $\parallel$ | $\mathtt{r_3}$ := y;<br>z := $\mathtt{r_3}$;<br>x := 1 |

(TC7)

As noted by Jeffrey and Riely [15], the failure of this test "indicates a failure to validate the reordering of independent reads".

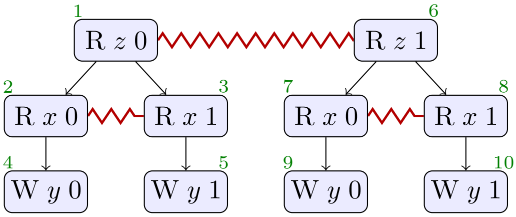

In the event structure of $T_1$ above, the justification relation is constructed according to Section 5. In particular, the rule for prepending reads (equation (4.1)) gives us $\{1,2\} \vdash_{T_1} 4$ and $\{1,3\} \vdash_{T_1} 5$ on the left-hand side, and $\{6,7\} \vdash_{T_1} 9$ and $\{6,8\} \vdash_{T_1} 10$ on the right. When composing the left and right sides, the coproduct rule (Section 4.2) makes four independent links, namely, $\{2\} \vdash_{T_1} 4$, $\{3\} \vdash_{T_1} 5$, $\{7\} \vdash_{T_1} 9$, and $\{8\} \vdash_{T_1} 10$. This is because, at the top level, for both branches, we can choose a write with the same label that is dependent on the same reads (plus the top ones on z). More precisely, on the left-hand side $C_1 = \{1,2\}$ is such that $C_1 \vdash_{T_1} 4$, and on the right-hand side $C_2 = \{6,7\}$ is such that $C_2 \vdash_{T_1} 9$. When the top events, 1 and 6 respectively, are removed, these contexts become isomorphic ($C_1[1] \cong C_2[6]$). Hence, $\{2\} \vdash_{T_1} 4$ and $\{7\} \vdash_{T_1} 9$, and $\{3\} \vdash_{T_1} 5$ and $\{8\} \vdash_{T_1} 10$.

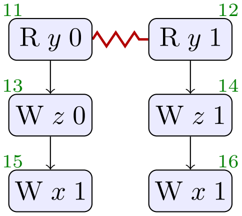

Now consider the event structure for the thread $T_2$. Here we have two independent writes, namely $\vdash_{T_2} (15 : \text{W } x\ 1)$ and $\vdash_{T_2} (16 : \text{W } x\ 1)$, arising in the coproduct from justifications $\{11\} \vdash_{T_2} (15 : \text{W } x\ 1)$ and $\{12\} \vdash_{T_2} (16 : \text{W } x\ 1)$. Notice that by definition (3), we do not add the writes 13 and 14 to the justification sets of any W $x$ 1, and because they write different values to z depending on the value of y, we have the dependencies $\{11\} \vdash_{T_2} 13$ and $\{12\} \vdash_{T_2} 14$.

When parallel composing, we connect the RF-edges that respect coherence. Thus we obtain the execution $\{16 \xrightarrow{\text{RF}} 8 \xrightarrow{\text{DP}} 10 \xrightarrow{\text{RF}} 12 \xrightarrow{\text{DP}} 14 \xrightarrow{\text{RF}} 6\}$, which is coherent, allowing the outcome with $\texttt{r}_1$, $\texttt{r}_2$ and $\texttt{r}_3$ all 1 as desired.

#### 6.3 Adding writes to justifications

In the definition of prepending writes (equation (3), condition (2)) we state that for any given justification, if there is an event in the justification set that is related via $\leq^{\mathcal{X}}$ with the write we are prepending, then that write must be in the justification set as well.

To see why we made this choice consider the following program,

```
x := 1;
r1 := y;
if(r1==0){
   x := 0; r2 := x; if(r2==1){z := 1}        || r3 := z;
} else {                                      || if(z==1){y := 1}
   r3 := x; if(r3==1){z := 1}
}
```

and its associated event structure,

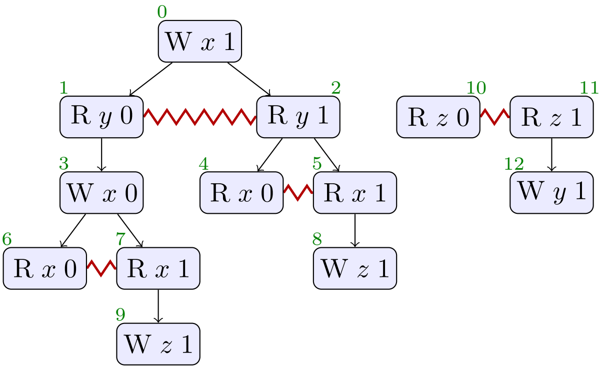

We focus on the interpretation of the left-hand side thread. In the equation (3), because $\{7\} \vdash 9$ and $3 \leq^{\mathcal{X}} 7$, the event $(3 : \text{W } x\ 0)$ gets inserted in the justification set, leading to the justification $\{3, 7\} \vdash 9$. On the other branch, up until the coproduct of the read on y, we have $\{5\} \vdash 8$. At this point, the justifications $\{7\} \vdash 9$ and $\{5\} \vdash 8$ are not lifted because 9 requires 3 as well. Event 3 may not be removed because of the condition in the write prepending rule. Without this condition 3 would not be necessary to justify 9, yielding the lifting of the link $\{5\} \vdash 8$. This would also cause the execution $\{0 \xrightarrow{\text{RF}} 5 \xrightarrow{\text{DP}} 8 \xrightarrow{\text{RF}} 11 \xrightarrow{\text{DP}} 12 \xrightarrow{\text{RF}} 2\}$ to be coherent due to the lack of a dependency between 2 and 5.

This execution is not sequentially consistent, but under SC, the program is race free. Without writes in justifications, the model would violate the DRF-SC property described in Section 5.2.

#### 6.4 Java memory model, Hotspot.

Finally, we discuss redundant read after read elimination, an optimisation performed by the Hotspot compiler but forbidden by the Java memory model. It is the first optimisation in the following sequence from Ševčík and Aspinall [30, Figure 5], used to demonstrate that the Java memory model is too strict, and unsound with respect to the observable behaviour of Sun's Hotspot compiler.

| $T_3$ | | $T_2$ | | $T_1$ |
|---|---|---|---|---|
| `r`$_2$ `:= y;`<br>`if(r`$_2$`== 1)`<br>$\{$`r`$_3$ `:= y; x := r`$_3\}$<br>else<br>$\{$`x := 1`$\}$ | $\longrightarrow$ | `r`$_2$ `:= y;`<br>`x := 1;` | $\longrightarrow$ | `x := 1;`<br>`r`$_2$ `:= y;` |

Consider the event structures of the unoptimised $T_3$ and optimised $T_1$.

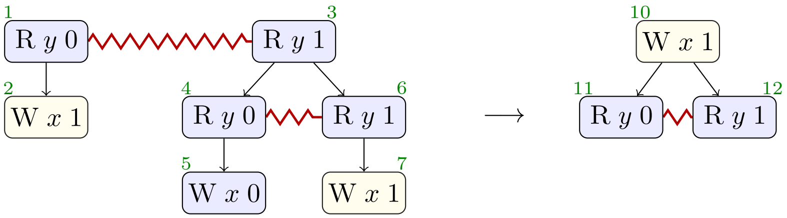

The optimisation removes the apparently redundant pair of reads $(4, 6)$, then reorders the now-independent write. This redundancy is represented in justification: when prepending the top read of `y` to the right-hand side of the event structure, the existing justification $6 \vdash 7$ is replaced by $3 \vdash 7$. When coproduct is applied, this matches with justification $1 \vdash 2$, leading to the independent writes $\vdash 2$ and $\vdash 7$. In a weak memory context however, a parallel thread could write a value to `y` between the two reads, thereby changing the value written to `x`. For this reason, we keep event 4 in the denotation and create the dependency edge $4 \xrightarrow{\text{DP}} 5$.

Despite exhibiting the same behaviour here, the denotations of $T_3$ and $T_2$ do not match. We establish that the optimisation is sound in any context in the next section.

### 7 Refinement

We have shown in Section 5.1 that our semantics enjoys a compositionality property: if we can prove that two programs have the same semantics (w.r.t set-theoretical equality) then they cannot be distinguished by any context. We also explained how equality is too strict, as it does not allow us to relate all programs that ought to be deemed semantically equivalent. Our Java Hotspot compiler example in Section 6 shows that the program $T_3$ is in practice optimised to $T_2$ and then to $T_1$. However, it is clearly not true that $[\![T_1]\!]_{n\ \rho\ \kappa}$ is a subset of $[\![T_2]\!]_{n\ \rho\ \kappa}$.

In this section we present a coarser-grained relation, which we call *refinement* ($\preccurlyeq$). This relation permits the optimisations we want, but remains sound w.r.t. the intuitive notion of observational equivalence, and that it is closed under context application in the same way as equality.

To show soundness we define *observational refinement* ($\preccurlyeq_{\mathrm{OBS}}$) which captures the intuitive notion of program equivalence: one program is a permissible optimisation of another if it does not increase the set of observable behaviours, defined here as changes to values of observed variables. The definition identifies related executions and compares the ordering of observable events, recognising that adding happens-before edges restricts behaviour. We then define a *refinement* relation and show this relation is a subset of observational refinement. This is formally stated in the following lemma:

**Lemma 2 (Soundness of Refinement ($\preccurlyeq \subseteq \preccurlyeq_{\mathbf{Obs}}$)).** *For all $P_1$ and $P_2$, if $[\![P_1]\!]^T_{n\ \rho\ \underline{\emptyset}} \preccurlyeq [\![P_2]\!]^T_{n\ \rho\ \underline{\emptyset}}$ then $[\![P_1]\!]^T_{n\ \rho\ \underline{\emptyset}} \preccurlyeq_{obs} [\![P_2]\!]^T_{n\ \rho\ \underline{\emptyset}}$*

Note that the refinement relation is defined over a tweaked version of the semantics, $[\![\cdot]\!]^T$, a variant of $[\![\cdot]\!]$ in which the registers are explicit in the event structure.

Finally we show $\preccurlyeq$ is compositional:

**Theorem 2 (Compositionality of Refinement ($\preccurlyeq$)).** *For all programs $P_1$ and $P_2$, and indexes $n$, if for all $\rho$, $[\![P_1]\!]^T_{n\ \rho\ \underline{\emptyset}} \preccurlyeq [\![P_2]\!]^T_{n\ \rho\ \underline{\emptyset}}$ then for all contexts $\mathcal{C}$, $\rho$, $\kappa$ and $\kappa'$ such that $\kappa \preccurlyeq \kappa'$ we have that $[\![\mathcal{C}[P_1]]\!]^T_{n\ \rho\ \kappa} \preccurlyeq [\![\mathcal{C}[P_2]]\!]^T_{n\ \rho\ \kappa'}$*

### 8 Showing implementability via IMM

In this section we show that our calculation of relaxed dependencies can easily be reused to solve the thin-air problem in other state-of-the-art axiomatic models, drawing the advantages of these models over to ours. In particular, we augment the IMM and RC11 models of Podkopaev et al. [26]. We adopt their language, given below. It covers C++ atomics, fences, fetch-and-add and compare-and-swap operations but excludes locks. Note that locks are implementable using compare and swap operations.

$$
\begin{aligned}
M &:= n \mid \mathtt{r} \\
B &:= M = M \mid B \wedge B \mid B \vee B \mid \neg B \\
T &::= \mathtt{skip} \mid \mathtt{r}\ \mathtt{:=}^{o_{\mathrm{R}}}\ \mathtt{x} \mid \mathtt{x}\ \mathtt{:=}^{o_{\mathrm{W}}}\ M \mid T_1\,;\,T_2 \\
&\quad \mid \mathbf{if}\,(B)\,\{P_1\}\{P_2\} \mid \mathbf{while}\,(B)\,\{P\} \\
&\quad \mid \mathbf{fence}^{o_{\mathrm{F}}} \mid \mathtt{r}\ \mathtt{:=}\ \mathbf{FADD}^{o_{\mathrm{R}},o_{\mathrm{W}}}_{o_{\mathrm{RMW}}}(\mathtt{x}, M) \\
&\quad \mid \mathbf{CAS}^{o_{\mathrm{R}},o_{\mathrm{W}}}_{o_{\mathrm{RMW}}}(\mathtt{x}, M, M)
\end{aligned}
\qquad
\begin{aligned}
P &::= T_1 \parallel \cdots \parallel T_n \\
o_{\mathrm{R}} &::= \mathit{rlx} \mid \mathit{acq} \\
o_{\mathrm{W}} &::= \mathit{rlx} \mid \mathit{rel} \\
o_{\mathrm{F}} &::= \mathit{acq} \mid \mathit{rel} \mid \mathit{acqrel} \mid \mathit{sc} \\
o_{\mathrm{RMW}} &::= \mathit{normal} \mid \mathit{strong}
\end{aligned}
$$

First we provide a model, written (for a program $P$) as $[\![P]\!]_{\mathsf{MRD+IMM}}$, that combines our relaxed dependencies to the axiomatic model of IMM, here written as $[\![P]\!]_{\mathsf{IMM}}$. We will make these definitions precise shortly. We then show that $[\![P]\!]_{\mathsf{MRD+IMM}}$ is weaker than $[\![P]\!]_{\mathsf{IMM}}$, making $[\![P]\!]_{\mathsf{MRD+IMM}}$ implementable over hardware architectures like x86-TSO, ARMv7, ARMv8 and Power. Secondly, we relax the RC11 axiomatic model by using our relaxed dependencies model MRD to create a new model $[\![P]\!]_{\mathsf{MRD\text{-}C11}}$, and show this model weaker than the RC11

model. We argue that the mathematical description of $[\![P]\!]_{\textsf{MRD-C11}}$ is lightweight and close to the C++ standard, it would therefore require minimal work to augment the standard with the ideas presented in this paper.

To prove implementability over hardware architectures we define a *pre-execution* semantics, where the relaxed dependency relation $\textsc{dp}$ is calculated along with the data and control dependencies from IMM. To combine our model with IMM, we redefine the $\textsc{ar}$ relation (we refer the reader to the IMM paper [26] for the details on $\textsc{ar}$) such that it is parametrised by an arbitrary relation which we put in place of the relations $(\mathsf{data} \cup \mathsf{ctrl})$. $\textsc{ar}(\mathsf{data} \cup \mathsf{ctrl})$ equals the original axiom $\textsc{ar}$ and $\textsc{ar}(\textsc{dp})$ is the same axiom where $\textsc{dp}$ is put in place of $\mathsf{data} \cup \mathsf{ctrl}$.

We define the executions in $[\![P]\!]_{\textsf{MRD+IMM}}$ as the maximal conflict-free sets such that $\textsc{ar}(\textsc{dp})$ is acyclic, and executions in $[\![P]\!]_{\textsf{IMM}}$ as the maximal conflict-free sets such that $\textsc{ar}(\mathsf{data} \cup \mathsf{ctrl})$ is acyclic.

#### 8.1 Implementability

We can now state and prove that the MRD model is implementable over IMM, which gives us that MRD is implementable over x86-TSO, ARMv7, ARMv8, Power and RISC-V by combining our result with the implementability result of IMM.

**Theorem 3 (MRD+IMM is weaker than IMM).** *For all programs $P$ by the* IMM *model,*

$$[\![P]\!]_{\textsf{MRD+IMM}} \supseteq [\![P]\!]_{\textsf{IMM}}$$

### 9 Modular Relaxed Dependencies in RC11: MRD-C11

We refer to the RC11 [18] model, as specified in Podkopaev et al. [26]. We call this model $[\![P]\!]_{\textsf{RC11}}$. While $[\![P]\!]_{\textsf{RC11}}$ forbids thin-air executions, it is not weak enough: it forbids common compiler optimisations by imposing that $(\sqsubseteq \cup \textsc{rf})$ is acyclic. We relax this condition by similarly replacing $\sqsubseteq$ with our relaxed dependency relation $\textsc{dp}$, this time calculated on our preserved program order relation $(\leq)$. We call this model $[\![P]\!]_{\textsf{MRD-C11}}$. Mathematically, this is done by imposing that $(\textsc{dp} \cup \textsc{rf})$ is acyclic.

At this point, we prove the following lemma:

**Lemma 3 (Implementability of MRD-C11).** *For all programs $P$,*

$$[\![P]\!]_{\textsf{MRD-C11}} \supseteq [\![P]\!]_{\textsf{RC11}}$$

To show this it suffices to show that there always exists $\textsc{dp} \subseteq \sqsubseteq$. This is straightforward by induction on the structure of $P$, observing that the only place where dependencies go against $\sqsubseteq$ is when hoisting a write in the coproduct case. However, in the same construction we always preserve the dependencies coming from the different branches of the structure which are, by inductive hypothesis, always agreeing with program order.

#### 9.1 MRD-C11 is DRF-SC

We show that MRD-C11 validates the DRF-SC theorem of the C++ standard [13, §6.8.2.1 paragraph 20].

**Theorem 4 (MRD-C11 is DRF-SC).** *For a program whose atomic accesses are all SC-ordered, if there are no SC-consistent executions with a race over non-atomics, then the outcomes of P under* MRD-C11 *coincide with those under SC.*

*Sketch proof.* In the absence of races and relaxed atomics, the no-thin-air guarantee of RC11 is made redundant by the guarantee of happens-before acyclicity shared by RC11 and MRD-C11. The result follows from this observation, lemma 3 and Theorem 4 from Lahav et al. [18].

### 10 On the Promising Semantics and WEAKESTMO

In this section we present examples that differentiate the Promising Semantics and WEAKESTMO from our MRD and MRD-C11 models.

First, we show that MRD correctly forbids the out-of-thin-air behaviour in the litmus test Coh-CYC from Chakraborty and Vafeiadis [11]. The test, given below, differentiates Promising and WEAKESTMO: only the latter avoids the outcome $r_1 = 3$, $r_2 = 2$ and $r_3 = 1$.

```
x := 2;                         ||   x := 1;
r1 := x; \\ 3                   ||   r2 := x; \\ 2
if(r1 != 2){y := 1}             ||   r3 := y; \\ 1
                                ||   if(r3 != 0){x := 3}
```

MRD correctly forbids this outcome: it identifies a dependency on the left-hand thread from the read of 3 from x to the write y := 1, and on the right-hand thread from the read of 1 from y to the write x := 3. The desired outcome then has a cycle in dependency and reads-from, and it is forbidden.

Chakraborty and Vafeiadis ascribe the behaviour to “a violation of coherence or a circular dependency”, and include specific machinery to WEAKESTMO that checks for global coherence violations at each step of program execution. These global checks forbid the unwanted outcome.

The Promising Semantics, on the other hand, can make promises that are not sensitive to coherence order, and therefore allows the above outcome erroneously.

In Coh-CYC, enforcing coherence ordering at each step in WEAKESTMO was enough to forbid the thin-air behaviour, but it is not adequate in all cases. The example below features an outcome that Promising and WEAKESTMO allow, and that MRD-C11 and MRD forbid. It demonstrates that cycles in dependency can arise without violating coherence in WEAKESTMO.

```
z := 1   ||   y := x   ||   if(z != 0){x := 1}{r0 := y; x := r0; a := r0}
```

The program is an adaptation[6] of a Java test, where the the unwanted outcome represents a violation of type safety [20]. Observing the thin-air behaviour where $\texttt{a} = 1$ in the adaptation above is the analogue of the unwanted outcome in the original test. If in the end $\texttt{a} = 1$, then the second branch of the conditional in the rightmost thread must execute. It contains a read of 1 from `y`, and a dependent write of `x := 1`. On the middle thread there is a read of 1 from `x`, and a dependent write of `y := 1`. These dependencies form the archetypal thin-air shape in the execution where $\texttt{a} = 1$. MRD correctly identifies these dependencies and the outcome is prohibited due to its cycle in reads-from and dependency.

The $\texttt{a} = 1$ outcome is allowed in the Promising Semantics: a promise can be validated against the write of `x := 1` in the true branch of the righthand thread, and later switched to a validation with $\texttt{x := r}_0$ from the false branch, ignoring the dependency on the read of `y`.

In the previous example, Coh-CYC, a stepwise global coherence check caused WEAKESTMO to forbid the unwanted behaviour allowed by Promising, but that machinery does not apply here. WEAKESTMO allows the unwanted outcome, and we conjecture that this deficiency stems from the structure of the model. Dependencies are not represented as a relation at the level of the global axiomatic constraint, so one cannot check that they are consistent with the dynamic execution of memory, as represented by the other relations. Adopting a coherence check in the stepwise generation of the event structure mitigates this concern for Coh-CYC, but not for the test above.

In contrast, MRD does represent dependencies as a relation, allowing us to check consistency with the RF relation here. The axiom that requires acyclicity of $(\textsc{dp} \cup \textsc{rf})$ forbids the unwanted outcome, as desired.

### 11 Evaluating MRD-C11 with the MRD-er tool

MRD-C11 is the first weak memory model to solve the thin-air problem for C++ atomics that has a tool for automatically evaluating litmus tests. Our tool, MRD-er, evaluates litmus tests under the base model, RC11 augmented with MRD, and IMM augmented with MRD. It has been used to check the result of every litmus test in this paper, together with many tests from the literature, including the Java Causality Test cases [7,11,15,16,18,25,26,27].

When evaluating whether a particular execution is allowed for a given test, a model that solves the thin-air problem must take other executions of the program into account. For example, the semantics of Pichon-Pharabod et al., having explored one execution path, may ultimately backtrack [25]. Jeffrey and Riely phrase their semantics as a two player game where at each turn, the player explores all forward executions of the program [15]. At each operational step, the Promising Semantics [16] has to run forwards in a limited local way to validate

[6] James Riely, Alan Jeffrey and Radha Jagadeesan provided the precise example presented here [28]. It is based on Fig. 8 of Lochbihler [20], and its problematic execution under Promising was confirmed with the authors of Promising.

that promised writes will be reached. The invisible events of Chakraborty et al. [11] are used to similar effect.

In MRD-C11, it is the calculation of justification that draws in information from other executions. This mechanism is localised, it avoids making choices about the execution that prune behaviours, and it does not require backtracking. MRD-C11 acts in a “bottom-up” fashion, and modularity ensures that justifications drawn from the continuation need not be recalculated. These properties have supported the development of MRD-er: automation of the model requires only a single pass across the program text to construct the denotation.

### 12 Discussion

Four recent papers have presented models that forbid thin-air values and permit previously challenging compiler optimisations. The key insight from these papers is that it is necessary to consider multiple program executions simultaneously. To do this, three of the four [15,25,11] use event structures, while the Promising Semantics [16] is a small-step operational semantics that explores future traces in order to take a step.

Although the Promising Semantics [16] is quite different from MRD, its mechanism for promising focuses on future writes, and MRD has parallels in its calculation of independent writes. Note also that both Promising's certification mechanism and MRD's lifting are thread-local.

The previous event-structure-based models are superficially similar to MRD, but all have a fundamentally different approach from ours: Pichon-Pharabod and Sewell [25] use event structures as the state of a rewriting system; Jeffrey and Riely [14,15] build whole-program event structures and then use a global mechanism to determine which executions are allowed; and Chakraborty et al. [11] transform an event structure using an operational semantics. In contrast, we follow a more traditional approach [33] where our event structures are used as the co-domain of a denotational semantics. Further, Jeffrey and Riely [14,15] and Pichon-Pharabod and Sewell [25] do not cover a significant subset set of C++ relaxed concurrency primitives.

MRD does not suffer from known problems with existing models. As noted by Kang et al. [16], the Pichon-Pharabod and Sewell model produces behaviour incompatible with the ARM architecture. The Jeffrey and Riely model forbids the reordering of independent reads, as demonstrated by Java Causality Test 7 (see Section 6.2). The Promising semantics allows the cyclic coherence ordering of the problematic Coh-CYC example [11]. WEAKESTMO allows the thin-air outcome in the Java-inspired test of Section 10. In all four cases MRD provides the correct behaviour.

MRD is also highly compatible with the existing C++ standard text. The DP relation generated by MRD can be used directly in the axiomatic model to forbid thin-air behaviour. We are working on standards text with the ISO C++ committee based on this work, and have a current working paper with them [5].

The notion in C++ that data-race free programs should not exhibit observable weak behaviours goes back to Adve and Hill [1], and formed the basis of the original proposal for C++ [10]. This was formalised by Batty et al. [8] and adopted into the ISO standard. Despite the pervasiveness of DRF-SC theorems for weak memory models, these have remained whole-program theorems that do not support breaking a program into separate DRF and racy components. Our DRF theorem for our denotational model demonstrates a limited form of modularity that merits further exploration.

Other denotational approaches to relaxed concurrency have not tackled the thin-air problem. Dodds et al. [12] build a denotational model based on an axiomatic model similar to C++. It forms the basis of a sound refinement relation and is used to validate data-structures and optimisations. Their context language is too restrictive to support a compositional semantics, and their compromise to disallow thin-air executions forbids important optimisations. Kavanagh and Brookes [17] provide a denotational account of TSO concurrency, but their model is based on pomsets and suffers from the same limitation as axiomatic models [7]: it cannot be made to recognise false dependencies.

*Future Work.* We envisage a generalised theorem that would, on augmentation with MRD, extend an axiomatic DRF-SC proof to a proof that applies to the augmented model.

The ISO have struggled to define `memory_order::consume` [13]. It is intended to provide ordering through dependencies that the compiler will not optimise away. The semantic dependency relation calculated by MRD identifies just these dependencies, and may support a better definition.

Finally, where we have used a global semantics to provide a full C++ model, it would be interesting to extend the denotational semantics to also cover all of C++, thereby allowing reasoning about C++ code in isolation from its context.

### 13 Conclusions

We have used the relatively recent insight that to avoid thin-air problems, a semantics should consider some information about what might happen in other program executions. We codify that into a modular notation of justification, leading to a semantic notion of independent writes, and finally of dependency (DP). We demonstrate the effectiveness of these concepts in three ways. One, we define a denotational semantics for a weak memory model, show it supports DRF-SC, and build a compositional refinement relation strong enough to verify difficult optimisations. Two, we show how to use DP with other axiomatic models, supporting the first optimal implementability proof for a thin-air solution via IMM, and showing how to repair the ISO C++ model. Three, we build a tool for executing litmus tests allowing us to check a large number of examples.
